# Impact of *Asaia* bacteria *on Leishmania major* development in sand flies: implications for vector control strategies

**DOI:** 10.1186/s13071-025-07075-5

**Published:** 2025-10-22

**Authors:** Marketa Stejskalova, Magdalena Jancarova, Katerina Pruzinova, Kristina Capova, Ilaria Varotto-Boccazzi, Sara Epis, Petr Volf

**Affiliations:** 1 Charles University, Prague, Czech Republic; 2 University of Milan, Milan, Italy

**Keywords:** Superinfection, *Asaia*, *Leishmania*, Sand flies

## Abstract

**Background:**

*Asaia* spp., bacteria originally isolated from tropical plants, have also been identified in various insect species, including blood-feeding ones. Their ability to colonize different host tissues and transmit vertically between generations makes these bacteria good candidates for paratransgenesis. However, most existing data derived from studies on mosquitoes and other important vectors, such as phlebotomine sand flies (Diptera: Psychodidae), remain understudied. In this study, we investigated the ability of wild-type *Asaia siamensis*, *Asaia krungthepensis*, and a genetically modified strain of *Asaia* expressing the *Wolbachia* surface protein (*Asaia*^WSP^) to colonize *Phlebotomus duboscqi*. In addition, we studied their vertical transmission and their interactions with *Leishmania major* during superinfection.

**Methods:**

*Phlebotomus duboscqi* females were provided with *Asaia* via a sugar meal. Bacterial presence and vertical transmission were assessed using both cultivation and polymerase chain reaction (PCR). In superinfection experiments, females were first offered sugar containing *Asaia*, followed by a blood meal infected with *Le. major*. The outcomes of superinfection were assessed by cultivation, PCR, and microscopically. Statistical analyses were performed using Fisher’s exact or Chi-squared tests.

**Results:**

All tested *Asaia* strains colonized the gut of *Ph. duboscqi*. Both *A. siamensis* and *A. krungthepensis* were vertically transmitted to the progeny via egg smearing. These bacteria did not affect the infection rate and intensity of *Le. major* infection on days 2 and 5 post blood meal (pbm). However, by day 8 pbm, both species significantly reduced *Le. major* infection intensity. Moreover, *A. krungthepensis* significantly increased the proportion of metacyclic forms. Interestingly *Asaia*^WSP^ did not have a significant effect on *Le. major* development in *Ph. duboscqi*.

**Conclusions:**

We demonstrated for the first time that *A. siamensis* and *A. krungthepensis* can infect *Ph. duboscqi* and be vertically transmitted to the next generation via egg smearing. These bacteria affect the late phase of *Le. major* infection, which could have important epidemiological consequences.

**Graphical Abstract:**

**Figure:**
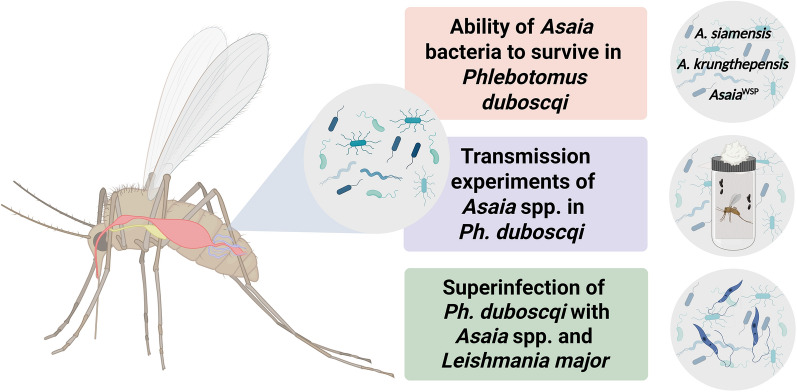

## Background

In arthropods, microorganisms play a crucial role in various aspects of their biology and life cycle, including nutrition, development, reproduction, immune response, behavior, defense against natural enemies, and speciation (reviewed by [1–3]). From a host’s perspective, interactions with microorganisms can be classified as pathogenic, when the microbes reduce their host fitness (i.e., antagonistic interactions); neutral, with no effect on the host fitness (i.e., commensal interactions); or beneficial, when the microbes enhance host fitness (i.e., mutualistic interactions) [4].

Acetic acid bacteria of the genus *Asaia*, originally isolated from tropical plants [5–7], are also able to colonize sugar-feeding insects from phylogenetically distant taxonomic groups [8–10]. Among blood sucking arthropods, *Asaia* sp. were detected in different mosquito and sand fly species [8, 11–16], reduviids [17], *Culicoides* spp. midges, and the tick *Haemaphysalis longicornis* [18]. The potential infectivity and risk of *Asaia* spp. to humans has also been raised as a concern, given that several reports have documented human infections, likely in the context of opportunistic infections, as *Asaia* does not circulate or establish stable transmission within the human population [19–25]. The most detailed insights into *Asaia* come from studies conducted on mosquitoes, where *Asaia* has been identified as a highly promising candidate for the paratransgenic control of vector-borne diseases [11, 26–28]. These bacteria colonize multiple mosquito tissues, including the gut, crop, salivary glands, and reproductive organs of both male and female mosquitoes [11, 12, 29, 30]. *Asaia* is transmitted between mosquitoes of the genus *Anopheles* during copulation, vertically from mother to offspring through egg-smearing, and horizontally during cofeeding [11, 12, 29]. Nevertheless, relatively little is known about the physiological role of this bacterium in mosquitoes. In addition to being a component of the microbiome, *Asaia* influences *Anopheles* mosquito larval development and adult longevity [31–33], causes transcriptomic changes [32, 33], and induces the activation of mosquito immunity [34]. However, the underlying mechanisms of these effects remain unknown.

Sand flies (Diptera: Psychodidae) are small nocturnal insects; both sexes feed on plant sap, nectar, and honeydew, while females also take blood meals on various vertebrate hosts. These insects have significant veterinary and medical importance as vectors of various pathogens infecting humans or domestic and wild animals. The most important sand-fly-borne pathogens are *Leishmania* spp., the causative agents of leishmaniasis (reviewed in [35]). There are three main clinical forms of the disease: (i) visceral, which is almost always fatal without treatment; (ii) cutaneous, which causes skin ulcers; and (iii) mucocutaneous, which leads to partial/total destruction of the nose, mouth, and throat mucous membranes. Every year, there are about 700,000 to a million new cases [36]. Although *Asaia* sp. have been repeatedly found in sand flies [13, 37], their effect on sand flies or transmitted pathogens is unknown.

As far as we know, this is the first study to examine in such detail the tripartite interactions between sand flies, *Asaia* sp., and *Leishmania* in vivo. Firstly, we infected females of *Phlebotomus duboscqi*, a vector of cutaneous leishmaniasis, with *Asaia krungthepensis* and *Asaia siamensis*, monitoring their survival and transmission to next generations of sand flies. Subsequently, we assessed the effect of these bacteria on the development of *Leishmania major*. In addition to using wild-type *Asaia* strains, we also investigated the effect of *Asaia* spp. expressing the *Wolbachia* surface protein (WSP) [28, 38].

## Methods

### Sand flies

The laboratory colony of *Phlebotomus duboscqi* (originally from Senegal) was maintained at the Laboratory of Vector Biology at Charles University in Prague under standard conditions [39]. Briefly, sand flies were kept at a temperature of 26 °C, a 14/10 light/dark photoperiod, and had access to a 50% sucrose solution ad libitum.

### *Leishmania*

*Leishmania major* LV561 (MHOM/IL/67/LRC-L137 Jericho II) was cultured at 23 °C in M199 medium (Sigma-Aldrich) supplemented with 10% fetal bovine serum (Gibco), 1% BME vitamins (Sigma-Aldrich), 2% filtered human urine, and amikacin (250 μg/mL).

### Bacteria

Four *Asaia* strains were used. Wild strains of *Asaia siamensis* (7132^T^) and *Asaia krungthepensis* (7333^T^) [6, 40] were provided by the Czech Collection of Microorganisms (Brno, Czech Republic). The genetically modified *Asaia* bacteria were kindly provided by Prof. Sara Epis from the University of Milan. The gene encoding the *Wolbachia* surface protein (WSP) was inserted into plasmid pHM4 and introduced into *Asaia* SF2.1, a wild-type strain derived from mosquitoes, resulting in the recombinant strain *Asaia*^WSP^. The control strain, *Asaia* pHM4, carried the empty plasmid [28]. *Asaia* spp. were cultivated on GLY medium (25 g/L glycerol [Lach-Ner s.r.o.], 10 g/L yeast extract, pH 5; Sigma-Aldrich). Agarose (20 g/L) was added when a solid medium was required to maintain colonies and detect bacteria during experiments. Kanamycin (100 µg/mL) was added to the medium to grow genetically modified bacteria carrying the plasmid*.*

### Bacteria preparation for sugar meal infection

Bacterial strains, regardless of species, were inoculated from agar plates into liquid GLY medium and incubated at 30 °C with shaking at 200 rpm until reaching an optical density corresponding to an infectious dose in the range of 1.8–4 × 10^8^ CFU/mL. Afterwards, the cultures were centrifuged twice at 6500 rpm for 5 min and washed with physiological saline solution to stop further bacterial growth. Separately, a 20% solution of cane sugar and distilled water was boiled in a microwave oven three times. The blue food coloring (AROMA a.s.) was added to the cooled solution, which was filtered using a sterile membrane filter (0.22 µM, Millipore Millex). The resulting bacterial pellets were mixed with a 20% sucrose solution, rather than the 50% sucrose used for colony maintenance, to minimize osmotic pressure. The mixture of pellet and sugar was stained with a food dye, which allowed visual confirmation of successful feeding.

### Experimental infection of sand flies with *Asaia* spp.

**Fig. 1:**
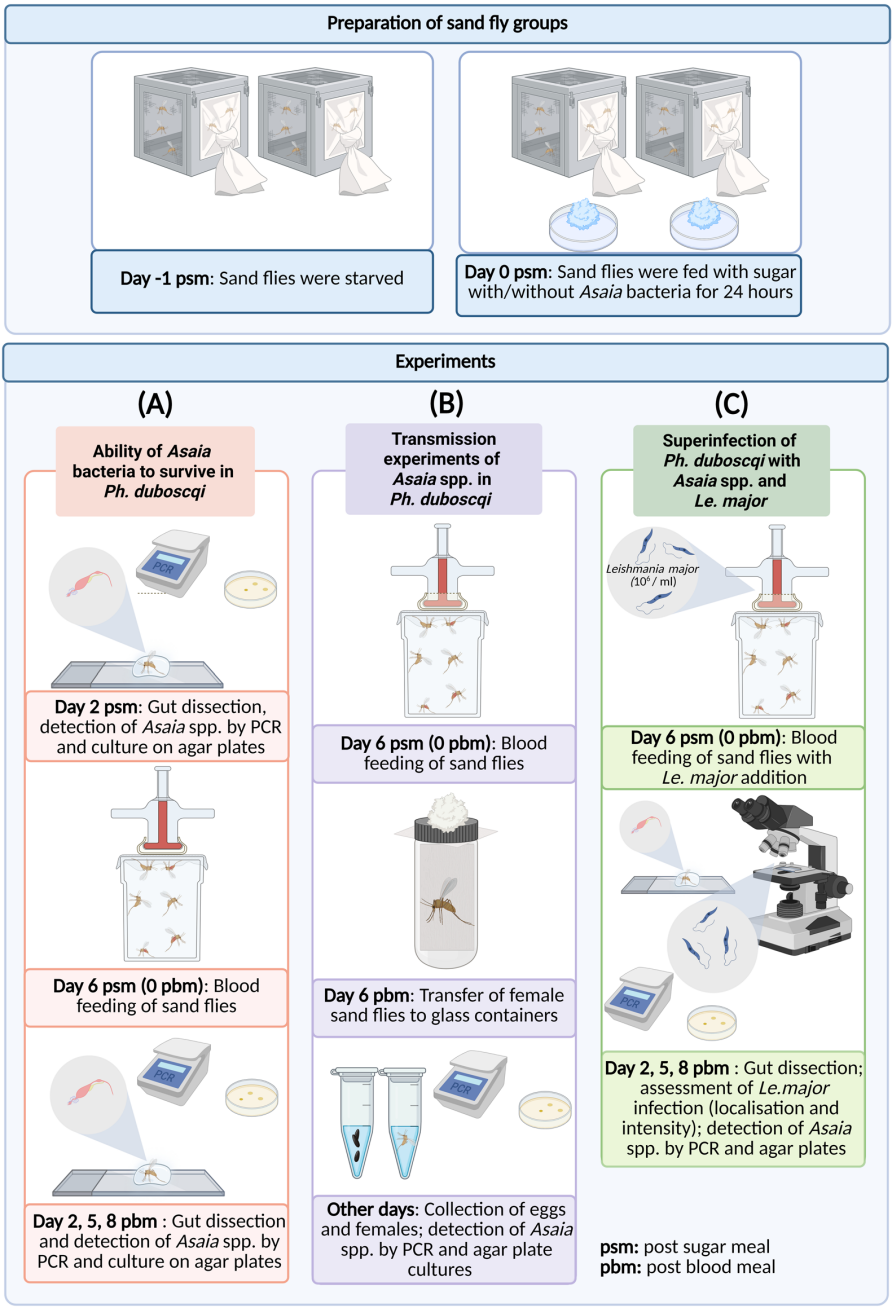
Experimental design of *Asaia* spp. colonization and subsequent superinfection with *Leishmania major* in female *Phlebotomus duboscqi*. In all experiments, females of *Phlebotomus duboscqi* were starved (−1 psm) and then on day 0 psm fed for 24 h on a control sugar solution or a sugar with bacteria of the genus *Asaia* (0 psm) (top panel). Three experiments were conducted on the group (**A**) ability of *Asaia* bacteria to survive in *Ph. duboscqi*, (**B**) transmission experiments of *Asaia* spp. in *Ph. duboscqi*, and (**C**) superinfection of *Ph. duboscqi* with *Asaia* spp. and *Le. major*. This schematic was created with BioRender.com

In each experiment, 120–150 *Ph. duboscqi* females (1–3 days old) were first starved for 24 h before the experiment and then offered a mixture of sugar solution with *Asaia* bacteria for another 24 h. Unfed females were removed from the cage, and infected females were maintained in standard conditions with access to a 50% sucrose solution (noninfected). On the second day post sugar meal (psm), 15–30 females per group were dissected to confirm the ability of *Asaia* spp. to survive in the digestive tract of *Ph. duboscqi* (Fig. 1A). Females were anesthetized on ice, and their legs were removed. To minimize external contamination, the surface of each sand fly was washed with distilled water containing a small amount of mild detergent, rinsed twice in distilled water, immersed in 70% ethanol for 15 s, and finally washed again with physiological saline. Then, females were dissected in a drop of phosphate-buffered saline (PBS) under a binocular microscope using a clamp and dissecting tools; the head was removed, and the gut (midgut plus hindgut with Malpighian tubules) was pulled out from the abdomen. Each gut was rinsed in a fresh drop of physiological saline, transferred individually into a microcentrifuge tube containing 100 µL of sterile physiological saline, and then manually homogenized with a pestle. From each homogenate sample, 10 µL was inoculated onto agar, and the remaining volume was used for PCR testing to verify both the presence and viability of the bacteria. A sample was considered positive if *Asaia* DNA was detected by PCR and bacterial growth was observed on solid medium (Fig. 1A).

On day 6 psm, *Ph. duboscqi* females were fed for 60–120 min with heat-inactivated sheep blood (LabMediaServis) through a chicken skin membrane on a glass feeder as described by Volf and Volfova [39] (day 0 post blood meal, i.e., day 0 pbm). On days 2, 5, and 8 pbm, 10–30 females from each group were dissected and processed as described above. The experiments were repeated three times for wild-type strains and twice for genetically modified bacteria.

### Molecular detection of *Asaia* spp.

DNA was isolated from homogenized samples using the High Pure PCR Template Preparation Kit (Roche) according to the manufacturer’s protocol. PCR reactions were performed in a total volume of 20 μL, containing 10 μL Emerald Amp GT PCR Master Mix Green (TaKaRa BIO INC.), 1 μL forward primer ASAFOR (5′-GCGCGTAGGCGGTTTACAC-3′; Sigma-Aldrich), 1 μL reverse primer ASAREV (5′-AGCGTCAGTAATGAGCCAGGTT-3′; Sigma-Aldrich), [11], and 2 μL template DNA and nuclease-free water to make up the final volume. The amplification protocol consisted of an initial denaturation at 95 °C for 2 min, followed by 30 cycles of denaturation at 95 °C for 30 s, annealing at 55 °C for 60 s, and extension at 72 °C for 60 s, with a final extension step at 72 °C for 4 min. The resulting PCR product was analyzed by agarose gel electrophoresis. PCR products were loaded onto a 1% agarose gel prepared from agarose powder (BioReagent, Sigma-Aldrich), 50× TAE electrophoresis buffer (Thermo Fisher Scientific), and SYBR Safe DNA Gel Stain (Thermo Fisher Scientific), which allows DNA visualization. For each sample, 10 μL of PCR product was loaded into the wells of the gel. The first and last wells were loaded with 10 µL of GeneRuler 100 bp DNA Ladder (Thermo Fisher Scientific) as a size standard. Electrophoresis was performed at 90–120 V for 20–55 min, depending on the size of the gel. The gel was visualized and photographed under blue light using a VILBER imaging system and then analyzed.

### *Asaia* spp. detection by cultivation

In total, 10 µL of homogenate were inoculated onto an agar plate with GLY medium and spread with a sterile glass stick over the entire surface to obtain separate colonies. The plates were then incubated in a thermostat for 48 h at 30 °C.

### Transmission experiments of *Asaia *spp. in *Ph. duboscqi*

To investigate the possibility of transovarial transmission of *Asaia* spp. in *Ph. duboscqi*, three experimental groups of 150 females were established. The females were fed on sugar with (1) *A. krungthepensis*, (2) *A. siamensis*, or (3) without bacteria (control group). On day 6 psm the sand flies were fed on the blood (0 pbm) (Fig. 1B). On day 6 pbm, individual females were placed into glass vials containing filter paper moistened with distilled water, and the inlet was covered with monofilament. Vials with females were placed in a plastic box, which was also lined with moist filter paper, and maintained at 26 °C and 70% relative humidity. Viability of *A. siamensis* and *A. krungthepensis* in females was controlled as described above. As soon as the female died, its carcass and laid eggs were separately sampled, homogenized, and tested by PCR, and inoculated on agar plates. Again, positive detection by PCR must be accompanied by positive cultivation. The experiment was repeated twice.

### Vertical transmission of *Asaia* spp.

After laying eggs, dead females were removed, and the eggs were divided into four groups: (1) nonhomogenized, unwashed clutches; (2) homogenized, washed clutches; (3) homogenized, unwashed clutches; and (4) nonhomogenized, washed clutches. The washing solution was prepared using a modified protocol by Poinar and Thomas [41]. Briefly, eggs were rinsed in distilled water, immersed in 500 µL of 70% ethanol for 5 min, and then in a 10% solution of sodium hypochlorite for the same period. After each chemical treatment, the eggs were rinsed in distilled water for 5 min to remove residual disinfectants. In addition, groups 2 and 3 were homogenized for 5 min using a homogenizer with an iron bead to assess whether *Asaia* bacteria were localized inside the eggs or merely adhered to their surface. The presence of *Asaia* in the homogenate was again evaluated by both PCR and agar plates. The experiments were repeated twice.

### Superinfection of *Ph. duboscqi* with *Asaia* spp. and *Leishmania major*

Groups of 120–150 *Ph. duboscqi* females (1–3 days old) were first infected with *Asaia* spp. using an infection dose of 1.8–4 × 10^8^ CFU/mL via sugar feeding. On day 6 psm/0 pbm, *Ph. duboscqi* females were fed for 60–120 min with heat-inactivated sheep blood (LabMediaServis), seeded with 1 × 10^6^ cells/mL promastigotes of *Le. major*, through a chicken skin membrane on a glass feeder. Non-blood-fed females were removed, and blood-fed ones were maintained at standard conditions. Between 15 and 30 *Ph. duboscqi* females were dissected on days 2, 5, and 8 post blood meal (pbm). Each female was washed as described above, and then the gut was dissected into a drop of PBS under a binocular microscope. The guts were examined microscopically for the presence, intensity, and localization of *Leishmania* infection. The intensity of infection was graded as (1) no infection, (2) weak (1–100 parasites/gut), (3) moderate (100–1000 parasites/gut), and (4) heavy (> 1000 parasites/gut) [42]. Three different localizations were distinguished, *Leishmania* either stayed in the abdominal midgut (AMG) or migrated anteriorly and also colonized the thoracic midgut (TMG) and stomodeal valve (SV). Subsequently, the dissected guts were rinsed from the microscope slide and coverslip using a pipette with 100 μL physiological saline solution, collected into a microtube, and homogenized. The homogenate was used for cultivation on agar plates and for PCR analysis to detect and confirm the presence of viable *Asaia* spp.

In addition, to evaluate *Leishmania* morphological forms present in the midgut, smears were made from *Le. major* positive guts using a coverslip. After drying, the sample was fixed with methanol and stained with Giemsa (Sigma-Aldrich).

Promastigotes of *Le. major* were classified as metacyclic forms when flagellum length was ≥ 2 times body length, leptomonad forms when flagellum length was < 2 times body length and body length was < 14 μm, and elongated nectomonads when flagellum length was < 2 times body length and body length was ≥ 14 μm according to [43]. The experiments were repeated three times for wild-type strains and twice for genetically modified bacteria (Fig. 1C).

## Statistical evaluation

The data were tested in StudioR (http://cran.r-project.org) [44]; owing to the type of data, Fisher’s exact or Chi-squared tests were used to assess the statistical significance.

## Results

### Colonization of *Ph. duboscqi* with *Asaia* spp.

**Fig. 2:**
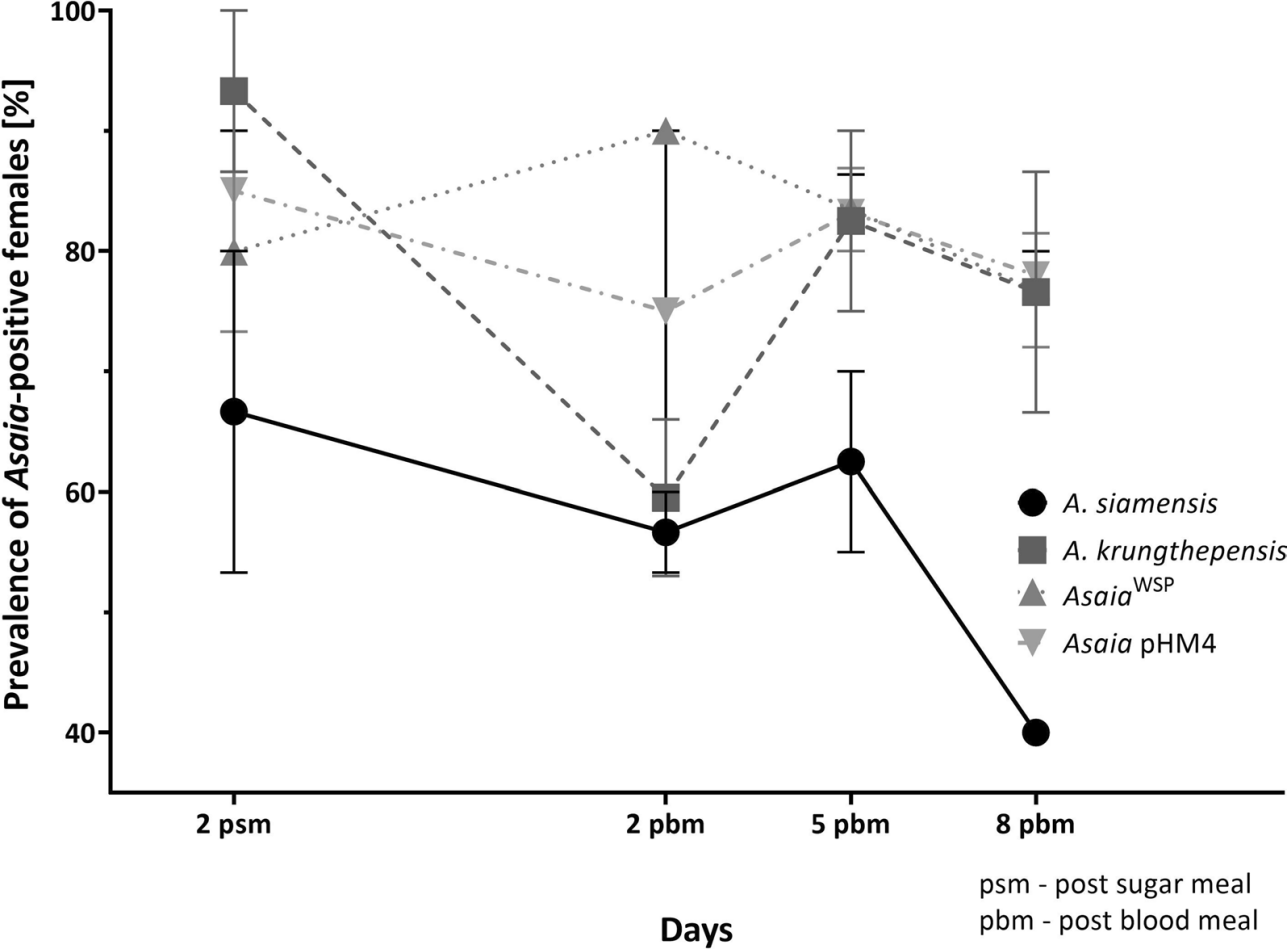
Prevalence of *Asaia*-positive females over time. *Asaia* strains (*A. siamensis*, *A. krungthepensis*, *Asaia*^WSP^, and *Asaia* pHM4) were tested for colonization of *Ph. duboscqi* midgut on various days post sugar meal (psm) and post blood meal (pbm). The data are shown as prevalence with the standard error of the mean (SEM), which originates from two independent experiments for genetically modified *Asaia* and three independent experiments for wild-type *Asaia*

All four *Asaia* strains tested (*A. siamensis*, *A. krungthepensis*, *Asaia*^WSP^, and *Asaia* pHM4) successfully colonized the gut of *Ph. duboscqi* following both the sugar meal (psm) and the subsequent blood meal (pbm) throughout the experimental period (Fig. 2). The lowest colonization was observed for *A. siamensis*, with infection rates of 66.7%, 56.7%, 62.5%, and 40% on day 2 psm and days 2, 5, and 8 pbm, respectively. *Asaia krungthepensis* showed a high initial infection rate on day 2 psm (93.3%), which dropped to 60% on day 2 pbm but increased again to nearly 80% on days 5 and 8 pbm, following blood defecation. A similar trend was observed for *Asaia* pHM4, though with slightly lower infection rates ranging from 71% to 85%. In contrast, *Asaia*^WSP^ maintained relatively stable and high infection rates, varying between 77% and 90% across all tested time points (Fig. 2).

### Vertical transmission of wild-type *Asaia* spp.

Out of 128 females infected with *A. siamensis*, 78 were positive and laid eggs, yielding 28 positive clutches. The highest positivity (80%) was detected in egg clutches laid on day 9 pbm (Fisher’s exact test, *P* = 0.001). Out of 128 females infected with *A. krungthepensis,* 69 were positive and laid eggs, and 41 of the clutches were infected. The positivity of egg clutches (Table 1) did not differ significantly across various days (Fisher’s exact test, *P* = 0.824) but was significantly higher compared with *A. siamensis* (*χ*^2^ = 7.2168, df = 1, *P* = 0.007).

**Table 1:** Summary of the number of *Asaia* positive/negative egg clutches per day after blood meal (pbm) laid by *Asaia*-infected *Ph. duboscqi* females

	Day pbm	6	7	8	9	10	11	12	13	14	Total (%)
*A. siamensis*	Positive	4	6	0	8	4	2	2	2	0	28 (36%)
	Negative	16	6	6	2	2	8	0	6	4	50 (64%)
*A. krungthepensis*	Positive	5	7	5	5	3	5	3	4	4	41 (59%)
	Negative	2	6	2	7	4	2	2	1	2	28 (41%)

In both *Asaia* species the vertical transmission was through egg-smearing. Regardless of homogenization, all test groups without egg washing were PCR positive, while washed eggs were all negative.

### Leishmania major infection in *Ph. duboscqi* infected by wild-type *Asaia* spp.

**Fig. 3:**
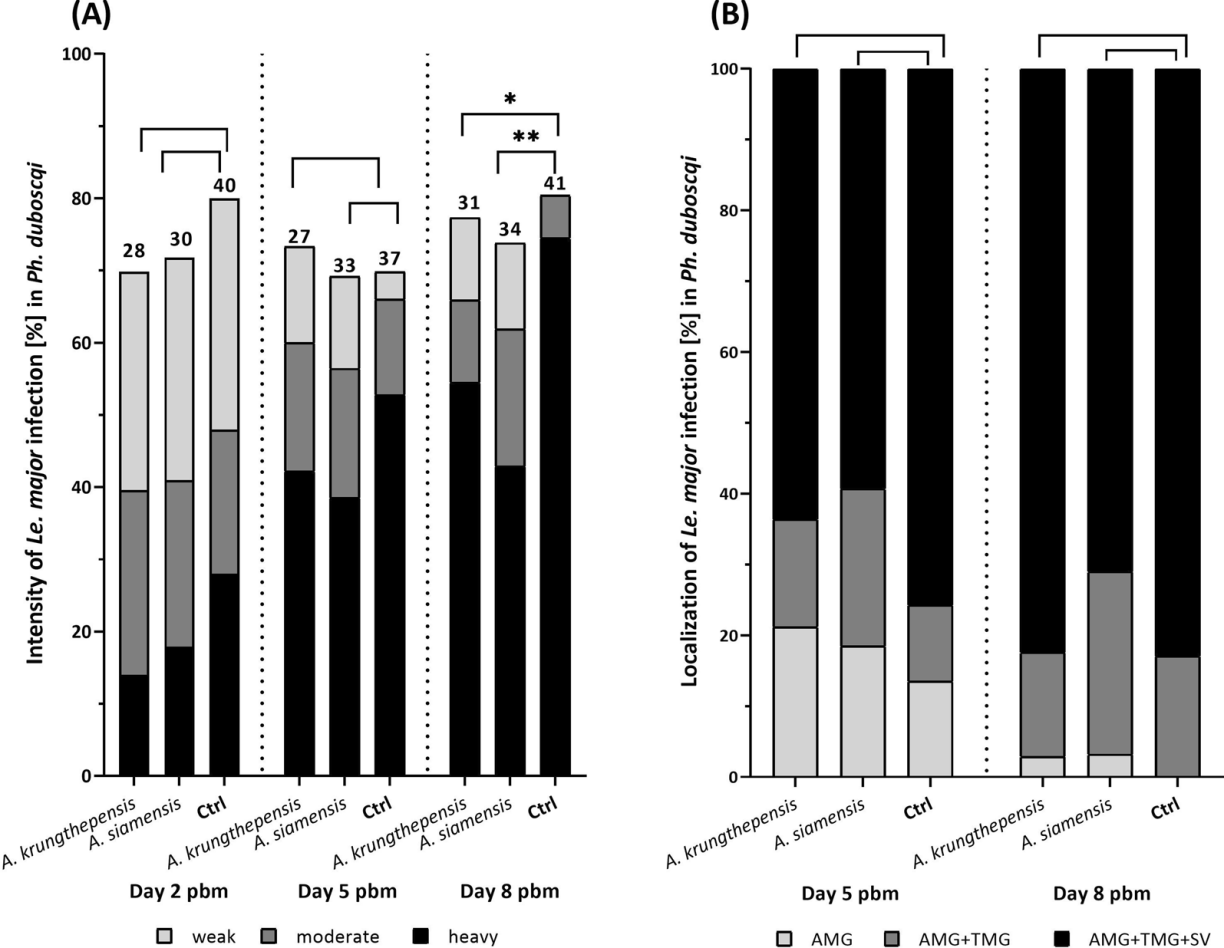
Intensity (**A**) and localization (**B**) of *Leishmania major* infection in *Phlebotomus duboscqi* females superinfected with wild-type *Asaia* strains. (**A**) The intensity of *Le. major* infection (%) in *Ph. duboscqi* females on days 2, 5, and 8 pbm. Three groups of females were compared: those infected with *A. siamensis* or *A. krungthepensis* and an *Asaia-*negative control group. Numbers above bars indicate the number of *Leishmania*–*Asaia*-positive females in each group. (**B**) Localization of infection on days 5 and 8 pbm. Only *Leishmania*-positive females from panel A were included; three categories of localization were distinguished: abdominal midgut (AMG), thoracic midgut (TMG), and stomodeal valve (SV). Data originate from three independent biological replicates

On day 2 pbm, i.e., before defecation, *Le. major* promastigotes were present in the endoperitrophic space, and the percentage of infected females ranged between 70% and 80% across all three experimental groups (Fig. 3A). Both the infection rates and parasite loads (i.e., infection intensities) did not significantly differ between females in the control group (without *Asaia*) and those colonized by *A. siamensis* (*χ*^2^ = 0.426, df = 1, *P* = 0.514; Fisher’s exact test, *P* = 0.729), nor between the control and the group colonized by *A. krungthepensis* (*χ*^2^ = 0.809, df = 1, *P* = 0.369). Similarly, on day 5 pbm (i.e., after defecation), no significant differences were observed among the groups in either the percentage of *Leishmania*-infected females or parasite loads (*A. siamensis*: *χ*^2^ = 0, df = 1, *P* = 1; *A. krungthepensis*: *χ*^2^ = 0.026, df = 1, *P* = 0.873; Fig. 3A) or intensity of infection (Fig. 3A) (*A. siamensis*: Fisher’s exact test, *P* = 0.297; *A. krungthepensis*: Fisher’s exact test, *P* = 0.318).

In late-stage infections (day 8 pbm), colonization by *A. siamensis* significantly reduced the intensity of *Le. major* infection (Fisher’s exact test, *P* = 0.002), but the percentage of infected females did not differ significantly (*χ*^2^ = 0.256, df = 1, *P* = 0.613). A similar effect was observed with *A. krungthepensis*, which also significantly reduced the incidence of heavy infections (Fisher’s exact test, *P* = 0.038), with no difference in percentage of infected females (*χ*^2^ = 0.014, df = 1, *P* = 0.905; Fig. 3A).

Regarding infection localization (Fig. 3B), neither *Asaia* strain had a significant effect on any of the days tested. For *A. siamensis*, no differences were found on day 5 pbm (Fisher’s exact test, *P* = 0.538) or day 8 pbm (Fisher’s exact test, *P* = 0.410). Similarly, for *A. krungthepensis*, no significant effect was observed on day 5 pbm (Fisher’s exact test, *P* = 0.705) or day 8 pbm (Fisher’s exact test, *P* = 0.839; Fig. 3B).

**Fig. 4:**
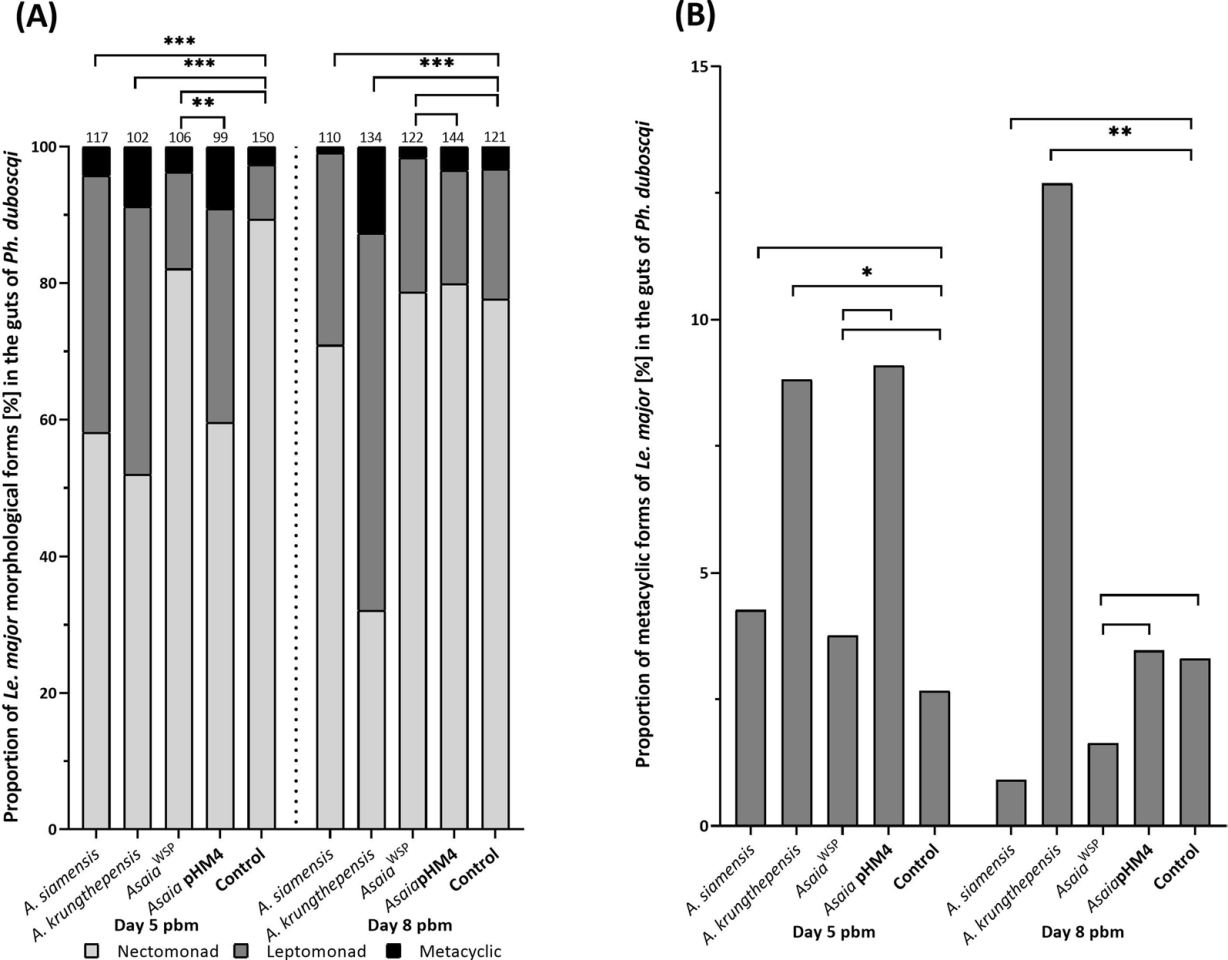
Morphological forms of *Leishmania major* in *Phlebotomus duboscqi* females superinfected with *Asaia* spp. (**A**) Proportions of three morphological forms of *Le. major* on days 5 and 8 pbm in sand fly females infected by two wild type strains (*A. siamensis* and *A. krungthepensis*), two genetically modified strains (*Asaia*^WSP^ and *Asaia* pHM4), and a control group (without *Asaia*). (**B**) Percentage of metacyclic promastigotes in these five groups. Numbers above the bars indicate the total number of measured *Le. major* morphological forms

As reported in Fig. 4A, three morphological forms of *Leishmania* (long nectomonads, leptomonads, and metacyclics) were identified on Giemsa-stained smears from *Ph. duboscqi* midguts colonized by *Asaia*. On day 5 pbm, colonization with *A. siamensis* and *A. krungthepensis* resulted in a significantly increased proportion of leptomonads at the expense of long nectomonads in both groups (Fisher’s exact test, *P* = 0.000). The proportion of metacyclic promastigotes was significantly higher in the group infected with *A. krungthepensis* (9%) compared with the *Asaia*-negative control group (3%). On day 8 pbm, females colonized with *A. krungthepensis* maintained the elevated proportion of leptomonads (Fisher’s exact test, *P* = 0.000), whereas no significant differences in the leptomonad-to-nectomonad ratio were observed between the *A. siamensis* group (Fisher’s exact test, *P* = 0.277) and the *Asaia*-negative control group (Fig. 4A). The proportion of metacyclic forms remained significantly higher in the *A. krungthepensis* group 17% compared with the control 4% (Fisher’s exact test, *P* = 0.011).

### *Leishmania major* infection in *Ph. duboscqi* infected by genetically modified *Asaia*

**Fig. 5:**
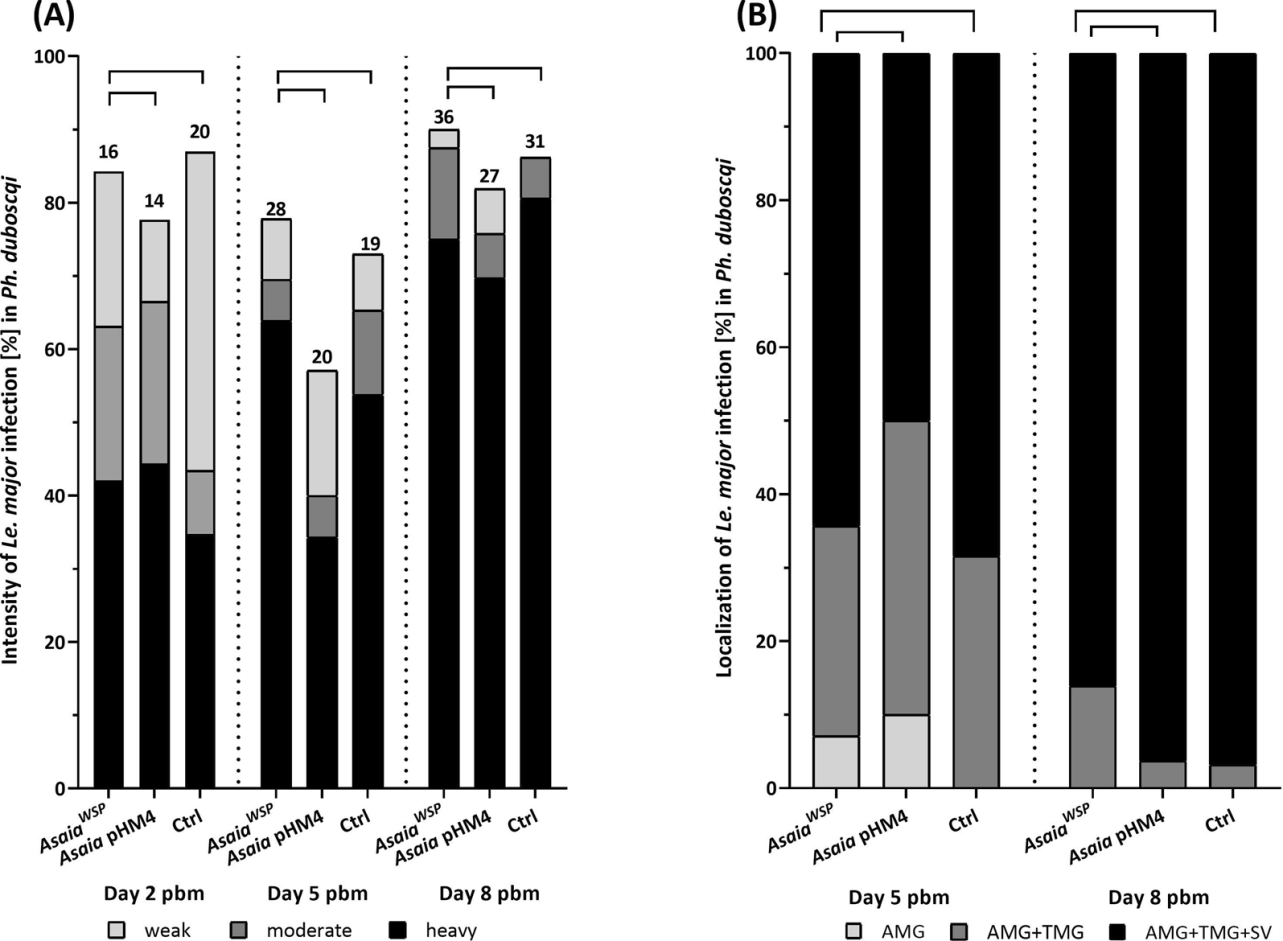
Intensity (**A**) and localization (**B**) of *Leishmania major* infection in *Phlebotomus duboscqi* females superinfected with genetically modified *Asaia* strains. **A**: The intensity of *Le. major* infection (%) in *Ph. duboscqi* females on days 2, 5, and 8 pbm. Three groups of females were compared: infected with *Asaia* pHM4, *Asaia*^WSP^, and an *Asaia-*negative control group. Numbers above bars indicate the number of *Leishmania*–*Asaia*-positive females in each group. **B** Localization of infection on days 5 and 8 pbm. Only *Leishmania*-positive females from panel A were included; three categories of localization were distinguished: abdominal midgut (AMG), thoracic midgut (TMG), and stomodeal valve (SV). Data originate from two independent biological replicates

On day 2 pbm, the *Leishmania* infection rates in sand fly groups superinfected with *Asaia*^WSP^ and *Asaia* pHM4 were 84% and 77%, respectively comparable to the infection rate observed in the control group without *Asaia* at 87% (Fisher’s exact test, *P* = 0.834; Fig. 5A). On days 5 and 8 pbm, *Leishmania* infection rates in females superinfected with *Asaia*^WSP^ and *Asaia* pHM4 fluctuated between 57 and 90%; however, no significant differences were detected compared to the *Asaia*-negative control (5 pbm: Fisher’s exact test, *P* = 0.1657; 8 pbm: *P* = 0.5731; Fig. 5A).

*Asaia*^WSP^ did not significantly affect the intensity of *Le. major* infection in any of the tested time points when compared either with the control group without bacteria (Fisher’s exact test, day 2 pbm: *P* = 0.131, day 5 pbm: *P* = 0.796, and day 8 pbm: *P* = 0.599) or with the group infected with *Asaia* pHM4 (Fisher’s exact test, day 2 pbm: *P* = 0.904, day 5 pbm: *P* = 0.078, and day 8 pbm: *P* = 0.565).

Similarly, there were no significant differences in *Le. major* localization among *Ph. duboscqi* sand flies infected with *Asaia*^WSP^ and *Asaia* pHM4 (Fisher’s exact test, day 5 pbm: *P* = 0.222 and day 8 pbm: *P* = 0.280), or in the control group without *Asaia* (Fisher’s exact test, day 5 pbm: *P* = 0.828 and day 8 pbm: *P* = 0.354; Fig. 5B). On day 5 pbm, sand flies infected with *Asaia*^WSP^ showed a higher proportion of leptomonad and fewer nectomonad forms compared with those infected with *Asaia* pHM4 (Fisher’s exact test, *P* = 0.005; Fig. 4A), in contrast to the comparison between *Asaia*^WSP^ and the group without *Asaia* (Fisher’s exact test, *P* = 0.409). On day 8 pbm, no significant differences were observed between the *Asaia*^WSP^ group and the control group without *Asaia* (Fisher’s exact test, *P* = 0.912), nor between *Asaia*^WSP^ and *Asaia* pHM4 (Fisher’s exact test, *P* = 0.780; Fig. 4B).

The proportions of metacyclic forms of *Le. major* in *Asaia*^WSP^-infected sand flies were similar to those in the *Asaia*-negative control group on day 5 (Fisher’s exact test, *P* = 0.721) and day 8 pbm (Fisher’s exact test, *P* = 0.446). Likewise, no significant differences were observed in the proportion of metacyclic promastigotes between *Asaia*^WSP^ and *Asaia* pHM4 groups on day 5 (Fisher’s exact test, *P* = 0.154) or day 8 pbm (Fisher’s exact test, *P* = 0.458).

## Discussion

In our experiments, all four *Asaia* species and strains tested were able to colonize the midgut of *Ph. duboscqi* and persisted throughout the observation period, after infection, and following blood feeding and subsequent defecations. However, their infection and survival dynamics differed over time.

The wild-type species, *A. siamensis* and *A. krungthepensis* showed similar colonization patterns, a decrease in infection rate following female blood feeding, a slight increase after defecation, and a subsequent decrease. A decline in microbial richness (i.e., the number of distinct operational taxonomic units) after blood feeding in sand flies was described by Kelly et al. [45], nevertheless, they showed that the richness was restored after defecation to a level seen with the sucrose-fed controls. At first glance, this seems inconsistent with our results, as the infection rate of *Asaia* continued to decline after defecation. However, while Kelly et al. [45] focused on overall species richness, our data specifically concern *Asaia.* There is a limited information on the behavior of *Asaia* in blood-feeding insects. Egyirifa and Akorli [46] described a decrease of *A. siamensis* in blood-fed *Anopheles gambiae*, and similar trends were observed in females of *Aedes aegypti*, *Aedes albopictus*, *Mansonia humeralis*, and *Asaia* sp. [47, 48].

Blood feeding alters the gut environment, inducing a shift in the microbiome abundance. Bacteria that thrive within the blood meal possess large genetic redox capacity to cope with oxidative and nitrosative stress associated with blood meal digestion [49]. It seems that wild-type species of *Asaia* are not among these species and may prefer non-blood-fed guts or other tissues. In our study, both *A. siamensis* and *A. krungthepensis* also infected the sand fly reproductive system, facilitating vertical transmission via contamination of the egg surface. Then the bacteria are ingested by the hatching larvae, a mechanism previously suggested for *Asaia* in mosquitoes [12] and leafhoppers [8].

In sand flies, similar vertical transmission was proven for *Psychodiella* gregarines [50]; however, to the best of our knowledge, this is the first study to rule out *Asaia* penetration through the egg membrane and confirm transmission via egg surface contamination.

It has been shown that native *Asaia* strains can activate mosquito immunity, potentially affecting pathogen development [34, 51]. *Asaia* introduction triggered mosquito immune responses that reduced *Plasmodium berghei* development in *Anopheles stephensi* [34], moreover, precolonization with *Asaia* sp. significantly reduced *Leishmania mexicana* populations in *Lutzomyia longipalpis* [16]. In our study, we tested the effect of *A. siamensis* and *A. krungthepensis* on the development of *Le. major*, the causative agent of zoonotic cutaneous leishmaniasis, in *Ph. duboscqi*. Both bacterial species significantly reduced parasite load during the late phase of infection. Notably, *A. krungthepensis* also altered the composition of the *Le. major* population, particularly increasing the proportion of metacyclic promastigotes. *Leishmania* development in sand flies proceeds through a series of promastigote morphotypes, among which, metacyclic promastigotes and possibly haptomonads are the main forms transmitted to mammalian hosts during blood feeding [52]. For transmission to mammals, both infection rate and infection intensity are important factors to consider. The presence of *Asaia* seems to modify this development, when both species decrease the intensity of infection in the late phase; nevertheless, *A. krungthepensis* stimulates metacyclogenesis.

Whether this effect is due to immune changes, production of anti-leishmanial molecules, microbiome alteration, or a combination of these factors remains to be determined and warrants further research. Nevertheless, the effect of *Asaia* spp. is less pronounced than that of the *Delftia tsuruhatensis* TC1 strain, which strongly inhibited *Le. major* development in *Ph. duboscqi*; importantly, *Leishmania*-infected sand flies fed with *D. tsuruhatensis* were significantly less able to transmit *Le. major* parasites and cause disease in mice [53].

Regarding the engineered strains, *Asaia* pHM4 and *Asaia*^WSP^, we showed that they retain the ability to colonize sand flies. Interestingly, especially in the case of *Asaia*^WSP^, abundance increased after the blood meal, contrasting with wild strains. Epis and collaborators [28] observed a similar pattern in *Aedes aegypti* mosquitoes. Despite Asaia^WSP^-induced activation of the host immune response in mosquitoes inhibiting *Dirofilaria immitis* in *Ae. aegypti* [28], in the present study, we found no impact on *Le. major* infection parameters in *Ph. duboscqi* or *Asaia* pHM4, and neither did *Asaia*^WSP^ affect the infection and development of *Le. major* in *Ph. duboscqi*.

*Wolbachia* has been repeatedly shown to activate the immune system in mosquitoes and negatively affect pathogen transmission [54–57]. However, its effect on sand flies remains unclear. *Wolbachia* have been repeatedly detected in sand flies [37, 58–60], but data on its impact on vector competence are still lacking. Nevertheless, Rosário and collaborators [61] detected *Wolbachia* and *Leishmania infantum* coinfections in 37% of *Nyssomyia whitmani* collected in the field, suggesting that co-existence in the same vector is possible. Similarly, an in vitro study showed that *Wolbachia* presence in sand fly cells did not significantly impact *Le. infantum* infection [62]. However, it should be noted that in our experimental model using *Asaia*^WSP^, the protein came from the filarial nematode *Dirofilaria immitis* [28], rather than an insect-associated strain. This exogenous WSP was intentionally used to induce a stronger immune response, but it may not accurately reflect the effects of naturally occurring *Wolbachia* infections in sand flies.

## Conclusions

We provided the first evidence that wild-type *Asaia* bacteria (*A. siamensis* and *A. krungthepensis*) can infect *Ph. duboscqi* and can be vertically transmitted via egg smearing. These bacteria reduce the late-phase infection of *Le. major*, which may have important epidemiological consequences and can be used for future vector control strategies. Unexpectedly, the *Asaia* strain expressing *Wolbachia* surface protein has no measurable effect on *Le. major* infection in this sand fly species.

## Data Availability

Data supporting the main conclusions of this study are included in the manuscript.

## References

[1] Shi H, Yu X, Cheng G. Impact of the microbiome on mosquito-borne diseases. Protein Cell. 2023;14:743–61. 37186167 10.1093/procel/pwad021 PMC10599646

[2] Motta EVS, Moran NA. The honeybee microbiota and its impact on health and disease. Nat Rev Microbiol. 2024;22:122–37. 38049554 10.1038/s41579-023-00990-3 PMC10998682

[3] Rajendran D, Vinayagam S, Sekar K, Bhowmick IP, Sattu K. Symbiotic bacteria: *Wolbachia*, midgut microbiota in mosquitoes and their importance for vector prevention strategies. Microb Ecol. 2024;87:154. 39681734 10.1007/s00248-024-02444-6 PMC11649735

[4] Lewis Z, Lizé A. Insect behaviour and the microbiome. Curr Opin Insect Sci. 2015;9:86–90. 32846714 10.1016/j.cois.2015.03.003

[5] Yamada Y, Katsura K, Kawasaki H, Widyastuti Y, Saono S, Seki T, et al. *Asaia bogorensis* gen nov., sp. nov, an unusual acetic acid bacterium in the alpha-Proteobacteria. Int J Syst Evol Microbiol. 2000;50:823–9. 10758893 10.1099/00207713-50-2-823

[6] Katsura K, Kawasaki H, Potacharoen W, Saono S, Seki T, Yamada Y, et al. *Asaia siamensis* sp. nov., an acetic acid bacterium in the *alpha-proteobacteria*. Int J Syst Evol Microbiol. 2001;51:559–63. 11321102 10.1099/00207713-51-2-559

[7] Bassene H, Niang EHA, Fenollar F, Doucoure S, Faye O, Raoult D, et al. Role of plants in the transmission of *Asaia* sp., which potentially inhibit the *Plasmodium* sporogenic cycle in *Anopheles* mosquitoes. Sci Rep. 2020;10:7144. 32346047 10.1038/s41598-020-64163-5 PMC7189373

[8] Crotti E, Damiani C, Pajoro M, Gonella E, Rizzi A, Ricci I, et al. *Asaia*, a versatile acetic acid bacterial symbiont, capable of cross-colonizing insects of phylogenetically distant genera and orders. Environ Microbiol. 2009;11:3252–64. 19735280 10.1111/j.1462-2920.2009.02048.x

[9] Li F, Hua H, Ali A, Hou M. Characterization of a bacterial symbiont *Asaia* sp. in the White-Backed planthopper, *Sogatella furcifera*, and its effects on host fitness. Front Microbiol. 2019;10:2179. 31620116 10.3389/fmicb.2019.02179 PMC6759652

[10] Woruba DN, Morrow JL, Reynolds OL, Chapman TA, Collins DP, Riegler M. Diet and irradiation effects on the bacterial community composition and structure in the gut of domesticated teneral and mature Queensland fruit fly, *Bactrocera tryoni* (Diptera: Tephritidae). BMC Microbiol. 2019;19:281. 31870300 10.1186/s12866-019-1649-6 PMC6929413

[11] Favia G, Ricci I, Damiani C, Raddadi N, Crotti E, Marzorati M, et al. Bacteria of the genus *Asaia* stably associate with *Anopheles stephensi*, an Asian malarial mosquito vector. Proc Natl Acad Sci U S A. 2007;104:9047–51. 17502606 10.1073/pnas.0610451104 PMC1885625

[12] Damiani C, Ricci I, Crotti E, Rossi P, Rizzi A, Scuppa P, et al. Mosquito-bacteria symbiosis: the case of *Anopheles gambiae* and *Asaia*. Microb Ecol. 2010;60:644–54. 20571792 10.1007/s00248-010-9704-8

[13] Akhoundi M, Bakhtiari R, Guillard T, Baghaei A, Tolouei R, Sereno D, et al. Diversity of the bacterial and fungal microflora from the midgut and cuticle of Phlebotomine sand flies collected in north-western Iran. PLoS ONE. 2012;7:e50259. 23226255 10.1371/journal.pone.0050259 PMC3511470

[14] Epis S, Gaibani P, Ulissi U, Chouaia B, Ricci I, Damiani C, et al. Do mosquito-associated bacteria of the genus *Asaia* circulate in humans? Eur J Clin Microbiol Infect Dis. 2012;31:1137–40. 21971818 10.1007/s10096-011-1419-3

[15] De Freece C, Damiani C, Valzano M, D’amelio S, Cappelli A, Ricci I, et al. Detection and isolation of the *α-proteobacterium Asaia* in *Culex* mosquitoes. Med Vet Entomol. 2014;28:438–42. 25387864 10.1111/mve.12045

[16] SantAnna MR, Diaz-Albiter H, Aguiar-Martins K, Al Salem WS, Cavalcante RR, Dillon VM, et al. Colonisation resistance in the sand fly gut *Leishmania* protects *Lutzomyia longipalpis* from bacterial infection. Parasit Vectors. 2014;7:329. 25051919 10.1186/1756-3305-7-329 PMC4112039

[17] Tarabai H, Floriano AM, Zima J, Filová N, Brown JJ, Roachell W, et al. Microbiomes of blood-feeding triatomines in the context of their predatory relatives and the environment. Microbiol Spectr. 2023;11:e0168123. 37289079 10.1128/spectrum.01681-23 PMC10433993

[18] Xiao J, Yao X, Guan X, Xiong J, Fang Y, Zhang J, et al. Viromes of *Haemaphysalis longicornis* reveal different viral abundance and diversity in free and engorged ticks. Virol Sin. 2024;39:194–204. 38360150 10.1016/j.virs.2024.02.003 PMC11074643

[19] Snyder RW, Ruhe J, Kobrin S, Wasserstein A, Doline C, Nachamkin I, et al. *Asaia bogorensis* peritonitis identified by 16S ribosomal RNA sequence analysis in a patient receiving peritoneal dialysis. Am J Kidney Dis. 2004;44:e15-17. 15264206 10.1053/j.ajkd.2004.04.042

[20] Tuuminen T, Heinäsmäki T, Kerttula T. First report of bacteremia by *Asaia bogorensis*, in a patient with a history of intravenous-drug abuse. J Clin Microbiol. 2006;44:3048–50. 16891542 10.1128/JCM.00521-06 PMC1594642

[21] Abdel-Haq N, Savaşan S, Davis M, Asmar BI, Painter T, Salimnia H. *Asaia lannaensis* bloodstream infection in a child with cancer and bone marrow transplantation. J Med Microbiol. 2009;58:974–6. 19502367 10.1099/jmm.0.008722-0

[22] Alauzet C, Teyssier C, Jumas-Bilak E, Gouby A, Chiron R, Rabaud C, et al. *Gluconobacter* as well as *Asaia* species, newly emerging opportunistic human pathogens among acetic acid bacteria. J Clin Microbiol. 2010;48:3935–42. 20826638 10.1128/JCM.00767-10 PMC3020812

[23] Juretschko S, Beavers-May TK, Stovall SH. Nosocomial infection with *Asaia lannensis* in two paediatric patients with idiopathic dilated cardiomyopathy. J Med Microbiol. 2010;59:848–52. 20339020 10.1099/jmm.0.019067-0

[24] Carretto E, Visiello R, Bardaro M, Schivazappa S, Vailati F, Farina C, et al. *Asaia lannensis* bacteremia in a ‘needle freak’ patient. Future Microbiol. 2016;11:23–9. 26674160 10.2217/fmb.15.126

[25] Mohammad A, Nik Hashim NHH. A rare case of persistent bacteremia caused by *Asaia* spp. in an infant. Cureus. 2024;16:e68577. 39371726 10.7759/cureus.68577 PMC11449687

[26] Shane JL, Grogan CL, Cwalina C, Lampe DJ. Blood meal-induced inhibition of vector-borne disease by transgenic microbiota. Nat Commun. 2018;9:4127. 30297781 10.1038/s41467-018-06580-9 PMC6175951

[27] Asgari M, Ilbeigikhamsehnejad M, Rismani E, Dinparast Djadid N, Raz A. Molecular characterization of RNase III protein of *Asaia* sp. for developing a robust RNAi-based paratransgensis tool to affect the sexual life-cycle of *Plasmodium* or *Anopheles* fitness. Parasit Vectors. 2020;13:42. 31996254 10.1186/s13071-020-3889-6 PMC6990573

[28] Epis S, Varotto-Boccazzi I, Crotti E, Damiani C, Giovati L, Mandrioli M, et al. Chimeric symbionts expressing a *Wolbachia* protein stimulate mosquito immunity and inhibit filarial parasite development. Commun Biol. 2020;3:105. 32144396 10.1038/s42003-020-0835-2 PMC7060271

[29] Damiani C, Ricci I, Crotti E, Rossi P, Rizzi A, Scuppa P, et al. Paternal transmission of symbiotic bacteria in malaria vectors. Curr Biol. 2008;18:R1087-1088. 19081038 10.1016/j.cub.2008.10.040

[30] Villegas LEM, Radl J, Dimopoulos G, Short SM. Bacterial communities of *Aedes aegypti* mosquitoes differ between crop and midgut tissues. PLoS Negl Trop Dis. 2023;17:e0011218. 36989328 10.1371/journal.pntd.0011218 PMC10085046

[31] Chouaia B, Rossi P, Epis S, Mosca M, Ricci I, Damiani C, et al. Delayed larval development in *Anopheles* mosquitoes deprived of *Asaia* bacterial symbionts. BMC Microbiol. 2012;12:S2. 22375964 10.1186/1471-2180-12-S1-S2 PMC3287513

[32] Mitraka E, Stathopoulos S, Siden-Kiamos I, Christophides GK, Louis C. *Asaia* accelerates larval development of *Anopheles gambiae*. Pathog Glob Health. 2013;107:305–11. 24091152 10.1179/2047773213Y.0000000106 PMC4001610

[33] Mancini MV, Damiani C, Short SM, Cappelli A, Ulissi U, Capone A, et al. Inhibition of *Asaia* in adult mosquitoes causes male-specific mortality and diverse transcriptome changes. Pathogens. 2020;9:380. 32429180 10.3390/pathogens9050380 PMC7281548

[34] Cappelli A, Damiani C, Mancini MV, Valzano M, Rossi P, Serrao A, et al. *Asaia* activates immune genes in mosquito eliciting an anti-*Plasmodium* response: implications in malaria control. Front Genet. 2019;10:836. 31608103 10.3389/fgene.2019.00836 PMC6774264

[35] Maroli M, Feliciangeli MD, Bichaud L, Charrel RN, Gradoni L. Phlebotomine sandflies and the spreading of leishmaniases and other diseases of public health concern. Med Vet Entomol. 2013;27:123–47. 22924419 10.1111/j.1365-2915.2012.01034.x

[36] World Health Organization. Leishmaniasis. 2023 https://www.who.int/news-room/fact-sheets/detail/leishmaniasis. Accessed 25 Jun 2025

[37] Karimian F, Koosha M, Choubdar N, Oshaghi MA. Comparative analysis of the gut microbiota of sand fly vectors of zoonotic visceral leishmaniasis (ZVL) in Iran; host-environment interplay shapes diversity. PLoS Negl Trop Dis. 2022;16:e0010609. 35853080 10.1371/journal.pntd.0010609 PMC9337680

[38] Varotto-Boccazzi I, Epis S, Arnoldi I, Corbett Y, Gabrieli P, Paroni M, et al. Boosting immunity to treat parasitic infections: *Asaia* bacteria expressing a protein from *Wolbachia* determine M1 macrophage activation and killing of *Leishmania* protozoans. Pharmacol Res. 2020;161:105288. 33160070 10.1016/j.phrs.2020.105288

[39] Volf P, Volfova V. Establishment and maintenance of sand fly colonies. J Vector Ecol. 2011;36:S1-9. 21366760 10.1111/j.1948-7134.2011.00106.x

[40] Yukphan P, Potacharoen W, Tanasupawat S, Tanticharoen M, Yamada Y. *Asaia krungthepensis* sp. nov., an acetic acid bacterium in the *α-Proteobacteria*. Int J Syst Evol Microbiol. 2004;54:313–6. 15023938 10.1099/ijs.0.02734-0

[41] Poinar GO, Thomas GM. Laboratory guide to insect pathogens and parasites. US: Springer; 1984.

[42] Myskova J, Votypka J, Volf P. *Leishmania* in sand flies: comparison of quantitative polymerase chain reaction with other techniques to determine the intensity of infection. J Med Entomol. 2008;45:133–8. 18283954 10.1603/0022-2585(2008)45[133:lisfco]2.0.co;2

[43] Sádlová J, Price HP, Smith BA, Votýpka J, Volf P, Smith DF. The stage-regulated HASPB and SHERP proteins are essential for differentiation of the protozoan parasite *Leishmania major* in its sand fly vector, *Phlebotomus papatasi*. Cell Microbiol. 2010;12:1765–79. 20636473 10.1111/j.1462-5822.2010.01507.x PMC3015063

[44] R Core Team. R: A language and environment for statistical computing R Foundation for Statistical Computing; 2018 https://www.r-project.org/. Accessed 17 Jun 2025

[45] Kelly PH, Bahr SM, Serafim TD, Ajami NJ, Petrosino JF, Meneses C, et al. The gut microbiome of the vector *Lutzomyia**longipalpis* is essential for survival of *Leishmania**infantum*. MBio. 2017;8:e01121-e1216. 28096483 10.1128/mBio.01121-16 PMC5241394

[46] Egyirifa RK, Akorli J. Two promising candidates for paratransgenesis, *Elizabethkingia* and *Asaia*, increase in both sexes of *Anopheles gambiae* mosquitoes after feeding. Malar J. 2024;23:45. 38347591 10.1186/s12936-024-04870-w PMC10863137

[47] Díaz S, Camargo C, Avila FW. Characterization of the reproductive tract bacterial microbiota of virgin, mated, and blood-fed *Aedes aegypti* and *Aedes albopictus* females. Parasit Vectors. 2021;14:592. 34852835 10.1186/s13071-021-05093-7 PMC8638121

[48] da Fonseca Meireles S, Ramalho MdeO, Montenegro H, do Nascimento Neto JF, da Silva JS, Cruz DLV, et al. Do the microbiota of larval breeding site and the blood meal influence the composition and diversity of bacterial communities in the midgut of *Mansonia humeralis* (Diptera: Culicidae) from the western Amazon? Braz J Microbiol. 2025;56:913–25. 39932664 10.1007/s42770-025-01623-y PMC12095781

[49] Wang Y, Iii TMG, Kukutla P, Yan G, Xu J. Dynamic gut microbiome across life history of the malaria mosquito *Anopheles gambiae* in Kenya. PLoS ONE. 2011;6:e24767. 21957459 10.1371/journal.pone.0024767 PMC3177825

[50] Lantova L, Volf P. The development of *Psychodiella sergenti* (Apicomplexa: Eugregarinorida) in *Phlebotomus sergenti* (Diptera: Psychodidae). Parasitol. 2012;139:726–34. 10.1017/S0031182011002411 PMC3332534 22313575

[51] Capone A, Ricci I, Damiani C, Mosca M, Rossi P, Scuppa P, et al. Interactions between *Asaia*, *Plasmodium* and *Anopheles*: new insights into mosquito symbiosis and implications in malaria symbiotic control. Parasit Vectors. 2013;6:182. 23777746 10.1186/1756-3305-6-182 PMC3708832

[52] Catta-Preta CMC, Ghosh K, Sacks DL, Ferreira TR. Single-cell atlas of *Leishmania* development in sandflies reveals the heterogeneity of transmitted parasites and their role in infection. Proc Natl Acad Sci U S A. 2024;121:e2406776121. 39700146 10.1073/pnas.2406776121 PMC11670217

[53] Cecilio P, Rogerio LA, D Serafim T, Tang K, Willen L, Iniguez E, et al. *Leishmania* sand fly-transmission is disrupted by *Delftia tsuruhatensis* TC1 bacteria. Nat Commun. 2025;16:3571. 40341020 10.1038/s41467-025-58769-4 PMC12062286

[54] Moreira LA, Iturbe-Ormaetxe I, Jeffery JA, Lu G, Pyke AT, Hedges LM, et al. A *Wolbachia* symbiont in *Aedes aegypti* limits infection with dengue, Chikungunya, and *Plasmodium*. Cell. 2009;139:1268–78. 20064373 10.1016/j.cell.2009.11.042

[55] Walker T, Johnson PH, Moreira LA, Iturbe-Ormaetxe I, Frentiu FD, McMeniman CJ, et al. The wMel *Wolbachia* strain blocks dengue and invades caged *Aedes aegypti* populations. Nature. 2011;476:450–3. 21866159 10.1038/nature10355

[56] Aliota MT, Walker EC, Yepes AU, Velez ID, Christensen BM, Osorio JE. The wMel strain of *Wolbachia* reduces transmission of Chikungunya virus in *Aedes aegypti*. PLoS Negl Trop Dis. 2016;10:e0004677. 27124663 10.1371/journal.pntd.0004677 PMC4849757

[57] Vandana V, Dong S, Sheth T, Sun Q, Wen H, Maldonado A, et al. *Wolbachia* infection-responsive immune genes suppress *Plasmodium falciparum* infection in *Anopheles stephensi*. PLoS Pathog. 2024;20:e1012145. 38598552 10.1371/journal.ppat.1012145 PMC11034644

[58] Bordbar A, Soleimani S, Fardid F, Zolfaghari MR, Parvizi P. Three strains of *Wolbachia pipientis* and high rates of infection in Iranian sandfly species. Bull Entomol Res. 2014;104:195–202. 24484966 10.1017/S0007485313000631

[59] Lozano-Sardaneta YN, Marina CF, Torres-Monzón JA, Sánchez-Cordero V, Becker I. Molecular detection of *Wolbachia* and *Bartonella* as part of the microbiome of phlebotomine sand flies from Chiapas, Mexico. Parasitol Res. 2023;122:1293–301. 37055642 10.1007/s00436-023-07829-z PMC10172221

[60] Torres-Llamas A, Díaz-Sáez V, Morales-Yuste M, Ibáñez-De Haro P, López-López AE, Corpas-López V, et al. Assessing *Wolbachia* circulation in wild populations of phlebotomine sand flies from Spain and Morocco: implications for control of leishmaniasis. Parasit Vectors. 2025;18:155. 40287743 10.1186/s13071-025-06771-6 PMC12032678

[61] Rosário AAdo, Dias-Lima AG, Lambert SM, Souza BMPdaS, Bravo F. Identification and molecular characterization of *Wolbachia* strains and natural infection for *Leishmania* sp. in neotropical *Phlebotominae* (Diptera: Psychodidae) species, leishmaniasis vectors. Acta Trop. 2022;235:106624. 35914568 10.1016/j.actatropica.2022.106624

[62] da Silva Gonçalves D, Iturbe-Ormaetxe I, Martins-da-Silva A, Telleria EL, Rocha MN, Traub-Csekö YM, et al. *Wolbachia* introduction into *Lutzomyia longipalpis* (Diptera: Psychodidae) cell lines and its effects on immune-related gene expression and interaction with *Leishmania infantum*. Parasit Vectors. 2019;12:33. 30646951 10.1186/s13071-018-3227-4 PMC6332621

